# Neural and Behavioral Effects of an Adaptive Online Verbal Working Memory Training in Healthy Middle-Aged Adults

**DOI:** 10.3389/fnagi.2019.00300

**Published:** 2019-11-01

**Authors:** Mónica Emch, Isabelle Ripp, Qiong Wu, Igor Yakushev, Kathrin Koch

**Affiliations:** ^1^Department of Neuroradiology, School of Medicine, Klinikum Rechts der Isar, Technical University of Munich, Munich, Germany; ^2^TUM-Neuroimaging Center, Technical University of Munich, Munich, Germany; ^3^Graduate School of Systemic Neurosciences, Ludwig-Maximilians-Universität, Martinsried, Germany; ^4^Department of Nuclear Medicine, School of Medicine, Klinikum Rechts der Isar, Technical University of Munich, Munich, Germany

**Keywords:** task-fMRI, working memory training, active control group, verbal working memory, middle-aged adults, fronto-parietal activation, supramarginal gyrus, n-back task

## Abstract

Neural correlates of working memory (WM) training remain a matter of debate, especially in older adults. We used functional magnetic resonance imaging (fMRI) together with an n-back task to measure brain plasticity in healthy middle-aged adults following an 8-week adaptive online verbal WM training. Participants performed 32 sessions of this training on their personal computers. In addition, we assessed direct effects of the training by applying a verbal WM task before and after the training. Participants (mean age 55.85 ± 4.24 years) were pseudo-randomly assigned to the experimental group (*n* = 30) or an active control group (*n* = 27). Training resulted in an activity decrease in regions known to be involved in verbal WM (i.e., fronto-parieto-cerebellar circuitry and subcortical regions), indicating that the brain became potentially more efficient after the training. These activation decreases were associated with a significant performance improvement in the n-back task inside the scanner reflecting considerable practice effects. In addition, there were training-associated direct effects in the additional, external verbal WM task (i.e., HAWIE-R digit span forward task), and indicating that the training generally improved performance in this cognitive domain. These results led us to conclude that even at advanced age cognitive training can improve WM capacity and increase neural efficiency in specific regions or networks.

## Introduction

Working memory (WM) is a capacity-limited cognitive system which is responsible for not only temporally storing information but also manipulating it (Baddeley, 2010). Research on WM is well motivated by the fact that WM exhibits correlations with cognitive abilities such as fluid intelligence (Chooi, 2012), reading comprehension (Daneman and Carpenter, 1980), or mathematical problem solving (Wiley and Jarosz, 2012). Therefore, during the past decade there has been mounting interest in training designs aimed at improving our WM capacity. The most prominent target population of such cognitive interventions is the older demographic group, as it has been shown that WM capacity decreases with age (Park and Reuter-Lorenz, 2009; Pliatsikas et al., 2018). The present paper focuses on the investigation of verbal working memory (vWM) and its training-associated changes, since vWM has been less investigated as compared to visuo-spatial WM, and has a tremendous importance for the daily life. There have been some attempts to study the neural correlates of vWM. In a recently published paper we performed a systematic fMRI meta-analysis to explore the neural correlates of vWM (Emch et al., 2019). We found vWM was associated with brain activity within a fronto-parieto-cerebellar network as well as subcortical regions, such as parts of the basal ganglia.

There have been studies since 2002 aiming at investigating the effects of WM training, showing that WM can be improved when adequate training procedures are used (Klingberg et al., 2002; see von Bastian and Oberauer, 2014 for a review). A meta-analysis from last year demonstrated functional brain changes following WM training within different networks such as the dorsal attention and salience network, sensory areas, and striatum (Salmi et al., 2018). Moreover, a number of studies suggested that younger adults benefit more from training than older participants (Dahlin et al., 2008; Li et al., 2008), but behavioral plasticity effects have also been reported at advanced age (Borella et al., 2010), and even more advanced age (Buschkuehl et al., 2008). However, the lifelong potential for plasticity is far from being fully understood. Apart from these unresolved questions results of previous studies investigating the effects of WM training on brain activation are still quite heterogeneous, both with regard to location as well as direction (i.e., increases vs. decreases) of reported activation changes (Salmi et al., 2018). One important reason could be the methodological heterogeneity of the studies: Thus, the studies or study samples differed with regard to (1) age neglecting the fact that older populations present differences not only in brain function but also in behavioral performance compared to younger populations; (2) training tasks as well as intensity and duration of the trainings (Salmi et al., 2018) which can lead to less or stronger WM training effects (Jaeggi et al., 2008); thus, as summarized in a systematic review on the effects of WM training (von Bastian and Oberauer, 2014), increasing the total duration of the training seems to increase the probability that training effects carry over to cognitive processes not directly practiced by the training; (3) training conditions, i.e., in some studies participants performed the training sessions in the vicinity of the investigators in order to control whether the participants were doing the training (Jansma et al., 2001; Miró-Padilla et al., 2018), thus neglecting the observer’s paradox which could go along with a decrease in WM training effects. Given the decline in WM capacities with increasing age the decrease caused by the observer’s paradox might be even more pronounced in older populations; (4) participants’ motivation which had sometimes not been taken into account despite evidence of its impact on training gains especially in older populations (Carretti et al., 2011); and (5) the type of control condition (i.e., waiting control group without contact to the investigator vs. passive control group vs. active control group). Whereas the implementation of a “no contact” or “passive” control group allows retesting the effects arising from pre- and post-designs, an active control group additionally controls for expectancy effects and generic intervention effects, such as consequences of using a computer or having a regular training schedule (von Bastian and Oberauer, 2014). All these issues mentioned above should be considered when investigating the effects of a WM intervention program. Hence, taking the following aspects into consideration might counteract further result heterogeneity: The training should ideally be administered in the form of an online training unobserved by the investigator thus minimizing the negative impact of observation on performance while allowing to monitor participants and safeguarding regular participation (Kulikowski and Potasz-Kulikowska, 2016). As stated before, participants’ motivation should be taken into account since it has been shown to impact training gains (Linares et al., 2019). In order to motivate participants to continuously improve their WM capacity and complete the task, in the present study mean reaction time, and accuracy was reported at the end of each block. We are highly confident that this boosted participants’ motivation to improve from one session to the next.

We investigated a group of healthy middle-aged volunteers within a limited age range (i.e., 50–65 years). The inclusion of this age group should minimize the influence of relevant age-related changes, such as atrophy or amyloid plaques, while maximizing the usefulness of the training with regard to training gains. We also avoided the inclusion of subjects with cognitive impairment and cognitive complaints, which are preclinical cognitive declines associated with dementia (Knopman, 2012). The selected participants performed an adaptive online WM training task (i.e., n-back task with each session level adapted to the participant’s performance) in order to keep task demands and motivation on a high level. Regarding training extent little is known about the ideal training duration. The number and duration of training sessions varies strongly amongst the published studies up to now. Most trainings contain about 20 training sessions each lasting about 30 min, but only little systematic research investigated the optimal intensity and duration of WM training interventions. Given findings by Jaeggi et al. (2008) who reported dose-dependent training effects (i.e., the longer the training, the larger the effects) we decided for an above-average training extent comprising 32 sessions with a total duration of 8 weeks which should be sufficient to cause significant training-related effects. We employed an active control training demanding a low-level vWM training task for the verbal task (i.e., 1-back level), to make sure that training conditions were the same for both groups to control for the Hawthorne effect which describes an improvement in the participant’s performance in response to the increased attention to their behavior (Landsberger, 1958). Finally, to assess potential direct effects of the training, a vWM task was employed before and after the training (i.e., HAWIE-R digit span forward and backward), which is an established test to investigate this cognitive construct.

The aim of this study was to investigate the behavioral and neural changes following an adaptive online verbal WM training in healthy middle-aged participants between 50 and 65 years old. We expected to provide evidence for neural plasticity and/or improvement in behavioral performance in healthy adults within this specific age range.

## Materials and Methods

### Participants

**FIGURE 1:**
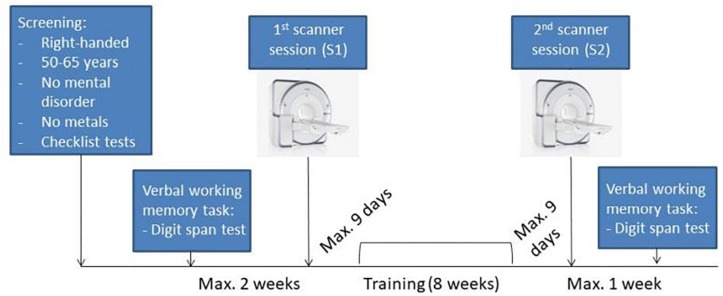
Experimental design. Scan image taken from © Siemens Healthcare GmbH, 2019.

Sixty-three subjects participated in the study. Six participants had to be excluded due to different reasons: one subject dropped out after the first session, two participants had clinically relevant alterations in brain structure, one volunteer moved more than 3 mm during the task-fMRI, one subject’s scanning data was not completely saved, and one participant was a training outlier. Therefore, the final sample contained fifty-seven healthy right-handed volunteers (28 male, 29 female) ranging between 50 and 65 years (mean age = 55.85 ± 4.24; mean years of education = 16.56 ± 3.14). Subjects were recruited via advertisements in the internet or newspaper. First, a telephone interview was conducted to assess the basic inclusion criteria: right handed, no mental disorder and presence of metal in the body. Afterward, the following diagnostic checklists were performed: the short form of the geriatric depression scale (GDS) (Yesavage et al., 1983), the mini-mental-status-test (MMST) (Folstein et al., 1975), the clock drawing test (Berit and Ove, 1998), and the M.I.N.I. International Neuropsychiatric Interview (Sheehan et al., 1998). Based on these screening, left-handed subjects, subjects with depression or other types of psychiatric disorders, and subjects with cognitive impairments were excluded from the study (see Figure 1 for study design).

Written informed consent was provided by each subject before the first session. Study participation was remunerated. Assignment of participants to one of the two groups (experimental or control group) occurred pseudo-randomly taking into account gender, age and years of education (YOE). The experimental group included 30 participants (mean age = 55.8 ± 4.3, 15 female, mean YOE = 16.96 ± 3.18), the control group consisted of 27 participants (mean age = 55.92 ± 4.25, 14 female, mean YOE = 16.11 ± 3.11). There were no significant differences between both groups regarding age, sex or YOE (*p* = 0.91, *p* = 0.89, *p* = 0.31, respectively). The study was approved by the Ethical Committee of the Klinikum Rechts der Isar and the Federal Office for Radiation Protection.

### Experimental Paradigm

#### Adaptive Online WM Training Task

We used the n-back task as WM training paradigm, in which letters are presented sequentially and the subject is asked to press a key whenever the current letter is identical to the one that appeared n-back positions earlier in the sequence. The active control group performed a low-level vWM training (i.e., stable level of verbal 1-back task). The vWM training of the experimental group was based on an adaptive online n-back paradigm comprising 9 blocks per session adapted from Jaeggi et al. (2010). In each block 6 targets were presented, meaning that the total number of possible hits was 54 per session. Both groups completed 32 training sessions with four sessions per week (i.e., 8 weeks in total) on their personal computers. Participants had the restriction of only performing one training session per day. In order to be able to analyze the training data we used the Inquisit software [Inquisit 5 (2016) retrieved from: https://www.millisecond.com], which is a precision software for online psychological experiments allowing the investigator to check for training participation and performance directly after each session. Each vWM training session started with a 1-back level and the level increased/decreased or stayed the same depending on the subject’s performance. Given a percentage of at least 90% correct answers, the n-back level increased by one in the next block. Given an accuracy level below 80%, the n-back level decreased by one. Otherwise, the n-back level remained the same. The maximum n-back level a participant could reach was 9. Both groups received a feedback at the end of each block (with regard to mean RT and percentage of correct answers). Both groups performed two different WM training modalities: verbal and visual n-back task. Given that the regions involved in verbal and visual WM processes are known to differ and considering that the visual n-back training differed significantly from the verbal training (i.e., the presented stimuli consisted of yellow abstract random shapes with low association value; the starting level was lower because of the unfamiliarity of the random shapes; and the active control group performed an attentional, i.e., X-back, visual online training) results of the visual training are reported elsewhere.

#### Task-fMRI Paradigm

**FIGURE 2:**
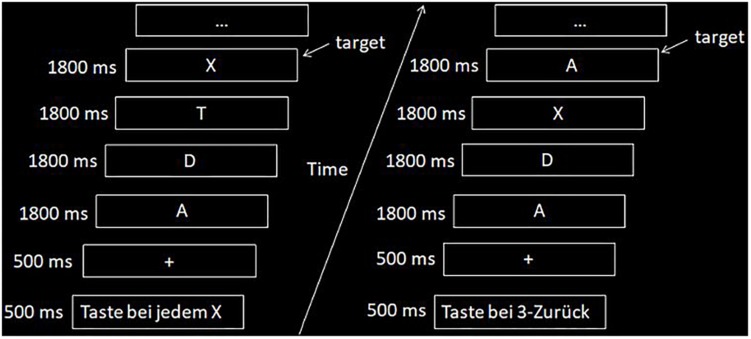
Example of the n-back fMRI task with the two conditions (i.e., left side, X-back; right side, 3-back).

In the scanner, subjects likewise performed a visual and a verbal n-back task. As already mentioned, visual WM results will be reported elsewhere. The WM paradigm was explained to the subjects before entering the scanner. In addition, subjects were asked to perform a short training version of the task to familiarize themselves with the stimulus presentation. Participants were allowed to repeat the practice task until they reported that they fully understood the task. The vWM task comprised the presentation of 26 capital white letters from the alphabet on a black background in the form of a block design. The whole task consisted of seven blocks of control condition (i.e., X-back task) and seven blocks of active task condition (i.e., 3-back task) presented in random order. Each condition lasted 45 s and consisted of 5 s of an instruction display indicating the following condition in German (3-back or X-back/0-back), 5 s of a fixation cross presentation, and 35 s of presentation of the letters (see Figure 2). Each block contained three possible hits giving a maximum of 21 possible hits per session and per condition. In the 3-back task any letter could be a target, in the X-back condition only the capital letter “X” was a target. The order of presentation with regard to verbal and visual n-back task was counterbalanced between the first and the second session. They did not receive a performance feedback after each block as in comparison for the training sessions.

#### Direct Effects

In order to investigate potential direct effects of the vWM training we asked participants to perform the HAWIE-R digit span sub-test (forward and backward version) (Molz et al., 2010) before and after the 32 training sessions. This test requires the subject to repeat up to nine numbers in the same order as read aloud by the examiner (forward version), and afterward in reverse serial order (backward version). Every item on the digit span test consists of two trials, each of which is scored with either 0 (incorrect) or 1 (correct). In case of at least one correct response, the examiner proceeds to read aloud the next-larger sequence of numbers. The task was explained beforehand and all participants practiced one short version of the task in order to familiarize themselves with the task. Performance assessment was based on the values of each subtest from the HAWIE-R and the test was orally presented with a rate of one number per second. The whole procedure lasted no more than 8 min. We hypothesized that if the participants successfully trained a specific process (i.e., vWM), they should demonstrate a significantly improved performance also in another test investigating the same process (i.e., HAWIE-R digit span).

### Behavioral Analysis

We used JASP^1^ and IBM SPSS Statistics software (Version 25 Armonk, New York, NY, United States) to analyze the fMRI behavioral data and the HAWIE-R test data. Two different statistical programs were employed to double-check the correctness of our results. Python version 3 was used to analyze the training data and scipy. stats was the package used for the statistical analyses. For the fMRI behavioral data we conducted two repeated-measures analyses of variance (ANOVAs) with Group (experimental group vs. control group) as between-subjects factor, Session (S1 vs. S2) as within-subject factor, and mean reaction time or *d’* values during each condition (3-back or X-back) as dependent variable. We selected *d’* instead of accuracy values [hits – false alarms (FA)] because this parameter takes the range for both components into account by calculating the relative proportion of hits minus FA (Haatveit et al., 2010; Meule, 2017). Higher values of *d’* means better performance whereas lower values of *d’* values means worse performance. We also performed a two-sample *t*-test between the active control and the experimental group at S1 (for the 3-back and X-back *d’* values as well as mean reaction time) to test whether there were any baseline differences between the groups. For the HAWIE-R subtest we likewise conducted repeated-measures ANOVAs with Group (experimental group vs. control group) as between-subjects factor and Session (S1 vs. S2) as within-subject factor.

For the training data, we analyzed the mean n-back level achieved in each session as well as the *d’* values. As data from the last three sessions of one subject in the experimental group were lost, we interpolated the missing data with her own previous training data with a forward linear method. *T*-tests comparing the first four and the last four sessions were performed to investigate whether there was a significant improvement in training performance in both groups.

### fMRI Acquisition

There were two scanner sessions: one immediately (i.e., no longer than 9 days) before the 8 weeks online training (S1) and another one immediately (i.e., no longer than 9 days) after the training (S2). The WM paradigm was presented using Presentation^®^ software (Version 18.0, Neurobehavioral Systems, Inc., Berkeley, CA, United States)^2^. The participants were able to see the task through a mirror fixed to the head coil which reflected the MRI-compatible screen. Participants were positioned supinely in the scanner. Their responses were collected via fORP 932 subject response package (Cambridge Research Systems). Participants held the button-box in their right hand and the emergency button in their left hand.

Images were acquired on a 3 T Biograph MR-PET Siemens scanner (Siemens, Erlangen, Germany), equipped with a 16-channel head coil at the Klinikum rechts der Isar, Munich, Germany. Specific cushions were used to prevent head movement. The imaging protocol included the following sequences: T1 MPRAGE, T2, FLAIR, DTI, echo-planar imaging (EPI) resting state, task-fMRI, and FDG-PET. Scan sessions lasted approximately 1 h. Results of the other sequences (i.e., DTI, resting-state fMRI, and FDG-PET) will be reported elsewhere. A high-resolution MPRAGE anatomical sequence was acquired with the following parameters: 160 slices; TR = 2300 ms; TE = 2.98 ms; flip angle = 9°; voxel size = 1.0 × 1.0 × 1.0; slice thickness = 1 mm; no gap; FOV = 256 mm; interleaved acquisition. Functional data were obtained using a gradient-echo T2^∗^-weighted EPI sequence with the following parameters: 237 slices; TR = 2700 ms; TE = 30 ms; flip angle = 90°; voxel size = 3.0 × 3.0 × 3.0; slice thickness = 3 mm; 0.6 mm gap; FOV = 192 mm; interleaved acquisition. The same sequences were used in S1 and S2.

### Image Preprocessing

Preprocessing as well as statistical analysis of fMRI data were conducted with SPM12 (Wellcome Department of Imaging Neuroscience, London, United Kingdom)^3^ in MATLAB v2018b. First, we performed head motion correction. Here the functional images were realigned and resliced to fit the mean functional image and then co-registered to the MPRAGE image using normalized mutual information. Movement was visually checked for each participant and participants moving more than 3 mm maximum displacement were not included in the final dataset. For the final dataset (*n* = 57) we calculated the root mean squared head position change (RMS movement) and converted the rotation parameters from degree to mm by calculating displacement on the surface of radius 50 mm to get the frame wise displacement (FD), as reported by Power et al. (2012, 2014). The FD is defined as the sum of absolute derivatives of these six parameters with the three rotational parameters converted to distance. There were no significant differences in both head motion parameters between both groups in S1 or S2 (see Table 1 for head movement parameters). Because subject motion not only degrades resting but also task-fMRI data, we censored some images to improve quality of task fMRI, as suggested in Siegel et al. (2014). We used a strict threshold of FD > 0.5 mm to censor the data since our study is based on a healthy cohort. We created a motion regressor taking into account the censored images. Then, we applied the Diffeomorphic Anatomical Registration Through Exponentiated Lie algebra (DARTEL) pipeline (Ashburner, 2007) to obtain a group specific structural template. We used it for segmentation and normalization to MNI space. Finally, data were smoothed using a 6 mm × 6 mm × 6 mm FWHM Gaussian Kernel.

**TABLE 1:** Head motion parameters.

		**Experimental group**	**Control group**	**Group differences *p*-value**
fMRI (S1)	Translation (mm)	0.109 ± 0.052	0.098 ± 0.063	0.505
	Rotation (rad)	0.045 ± 0.023	0.041 ± 0.027	0.512
fMRI (S2)	Translation (mm)	0.104 ± 0.051	0.09 ± 0.044	0.252
	Rotation (rad)	0.043 ± 0.022	0.036 ± 0.018	0.203

*Mean translation in mm ± *SD* and mean rotation in radius ± *SD* are presented for both groups and time points. *T*-tests were performed between groups at both time points.*

### Image Analyses

A general linear model at the single subject level was conducted to obtain the task activation contrasts of interest. The task design function was convolved with a canonical haemodynamic response function (HRF) and its time derivative, allowing for a slight temporal shift. Six motion realignment parameters and motion censor regressor (i.e., FD > 0.5 mm) were included as covariates of no interest. We used a high-pass filter of 220 s to the functional data to eliminate low-frequency components because the default filter (128 s) was not adequate for our design (i.e., a filter of 128 s would have removed parts of the task-related activation).

For the second level analysis we conducted a one-sample *t*-test to obtain areas activated during the n-back task (3-back > X-back level) in general. We also performed a two-sample *t*-test to examine whether there were differences at S1 between the experimental and the active control group. The longitudinal analyses were performed by assessing the interaction effects between Session (S1 vs. S2) and Group (experimental group vs. control group) using the factorial design in SPM. The statistical criterion was set at *p* < 0.05 false-discovery rate (FDR) corrected. In addition, the number of expected voxels per cluster was used an as an extent threshold.

## Results

### Behavioral Results

#### Cognitive Training

**FIGURE 3:**
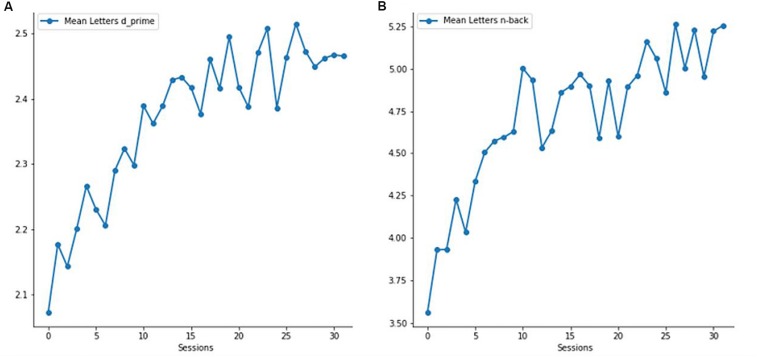
Verbal working memory (vWM) training performance of the experimental group. **(A)** Mean verbal *d’* values across all 32 sessions. **(B)** Mean verbal n-back level across all 32 sessions.

As is illustrated in Figure 3, the experimental group showed a significant improvement in both n-back level and *d’* values (both *p* < 0.001) when comparing performance between the first and the last four training sessions. In the control group, only *d’* values were analyzed, since the n-back level (i.e., 1-back level) stayed the same during all training sessions. Expectedly, *d’* values of the control group did not significantly differ between the first and last four training sessions (*p* = 0.184).

#### Direct Effects

**FIGURE 4:**
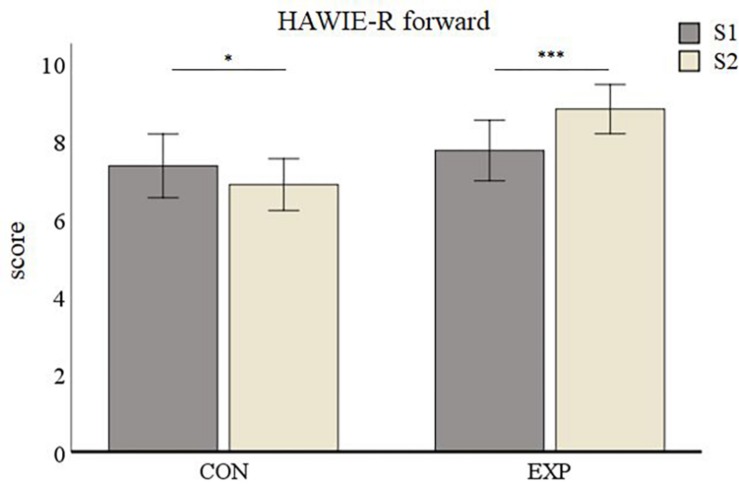
HAWIE-R subtest digit span (forward version) results. Data are presented as mean values ± SEM. ^∗^*p* < 0.05, ^∗∗^*p* < 0.01, and ^∗∗∗^*p* < 0.001; S1, first time point; S2, second time point; CON, control group; and EXP, experimental group.

The average HAWIE-R forward subtest values for the control group were 7.37 (*SD* = 0.41) at S1 and 6.89 (*SD* = 0.33) at S2. Those for the experimental group were 7.77 (*SD* = 0.39) at S1 and 8.83 (*SD* = 0.32) at S2. The repeated measures ANOVA on the HAWIE-R forward subtest showed a non-significant effect of Session [*F*_(__1_,_55__)_ = 2.46, *p* = 0.122] but a significant main effect for Group [*F*_(__1_,_55__)_ = 5.94, *p* = 0.018]. The interaction between Session and Group was significant [*F*_(__1_,_55__)_ = 17.248, *p* < 0.001, Figure 4]. *Post hoc* analyses revealed a performance decrease in the control group (*p* = 0.045) and a highly significant improvement in the experimental group (p < 0.001).

The average HAWIE-R backward subtest values for the control group were 6.85 (*SD* = 0.33) at S1 and 7.48 (*SD* = 0.43) at S2. Those for the experimental group were 6.73 (*SD* = 0.31) at S1 and 7.5 (*SD* = 0.41) at S2. The repeated measures ANOVA on the HAWIE-R backward subtest showed an effect of Session [*F*_(__1_,_55__)_ = 5.78, *p* = 0.02] and no effect of Group [*F*_(__1_,_55__)_ = 0.013, *p* = 0.91]. The interaction between Group and Session yielded no significant results [*F*_(__1_,_55__)_ = 0.056, *p* = 0.814].

#### Task-fMRI (*d*’)

**FIGURE 5:**
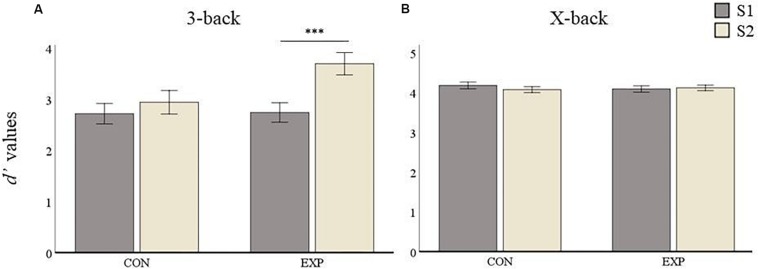
*D’* values results. Data are presented as mean values ± SEM. **(A)** 3-back condition results. **(B)** X-back condition results. ^∗^*p* < 0.05, ^∗∗^*p* < 0.01, ^∗∗∗^*p* < 0.001; S1, first time point; S2, second time point; CON, control group; and EXP, experimental group.

The comparison between experimental and active control group yielded no significant differences at baseline (S1) in any condition for *d’* values (i.e., 3-back: *p* = 0.864 and X-back: *p* = 0.124). The average 3-back *d’* values for the control group were 2.73 (*SD* = 0.53) at S1 and 2.96 (*SD* = 0.61) at S2. Those for the experimental group were 2.74 (*SD* = 0.51) at S1 and 3.69 (*SD* = 0.78) at S2 (see Figure 5A). The repeated measures ANOVA on the 3-back *d’* values showed a main effect for Session [*F*_(__1_,_55__)_ = 47.03, *p* < 0.001] and for Group [*F*_(__1_,_55__)_ = 10.33, *p* = 0.002] and, accordingly, the interaction between Session and Group was significant [*F*_(__1_,_55__)_ = 18.07, *p* < 0.001]. *Post hoc* analyses revealed no significant improvement in the control group (*p* = 0.06), but a highly significant improvement in the experimental group (*p* < 0.001).

For the X-back condition the control group had mean *d’* values of 4.18 (*SD* = 0.13) and 4.08 (*SD* = 0.19) at S1 and S2, respectively, whereas the experimental group had a mean of 4.10 (*SD* = 0.29) and 4.13 (*SD* = 0.21) at S1 and S2, respectively (see Figure 5B). The repeated measures ANOVAs for the X-back condition yielded no significant main effect for Session [*F*_(__1_,_55__)_ = 0.93, *p* = 0.34] or Group [*F*_(__1_,_55__)_ = 0.331, *p* = 0.567]. The interaction was also not significant [*F*_(__1_,_55__)_ = 2.74, *p* = 0.103] indicating no performance improvement for the X-back condition in any group after the training.

#### Task-fMRI (Mean Reaction Time)

**FIGURE 6:**
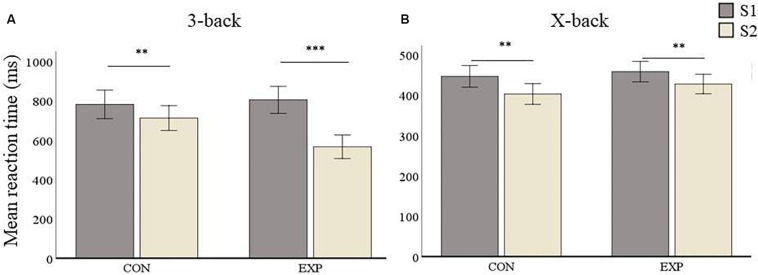
Mean RT (in ms) results. Data are presented as mean RT ± SEM. **(A)** 3-back condition results. **(B)** X-back condition results. ^∗^*p* < 0.05, ^∗∗^*p* < 0.01, ^∗∗∗^*p* < 0.001; S1, first time point; S2, second time point; CON, control group; and EXP, experimental group.

The comparison between experimental and active control group yielded no significant differences at baseline (S1) in any condition for mean reaction time (i.e., 3-back: *p* = 0.646 and X-back: *p* = 0.531). Mean reaction time (RT) 3-back for the control group was 782.7 ms (*SD* = 183.75) at S1 and 713.04 ms (*SD* = 172.31) at S2, whereas the experimental group had a mean RT of 805.71 ms (*SD* = 191.67) at S1 and 567.35 ms (*SD* = 155.75) at S2 (see Figure 6A). The repeated measures ANOVA conducted for 3-back mean reaction time showed a main effect of Session [*F*_(__1_,_55__)_ = 42.1, *p* < 0.001], no effect of Group [*F*_(__1_,_55__)_ = 2.3, *p* = 0.134], and a significant interaction between both factors [*F*_(__1_,_55__)_ = 12.63, *p* < 0.001]. *Post hoc* analyses revealed a significant improvement from S1 to S2 in the control group (*p* = 0.0017) as well as in the experimental group (*p* < 0.001, see Figure 6A).

In the X-back condition, the control group had a mean RT of 446.93 ms (*SD* = 72.95) at S1 and a mean RT of 403.21 ms (*SD* = 72.32) at S2, whereas mean RT in the experimental group was 458.62 ms (*SD* = 66.3) at S1 and 428.06 (*SD* = 60.53) at S2 (see Figure 6B). The repeated measures ANOVA for X-back showed a main effect of Session [*F*_(__1_,_55__)_ = 22.51, *p* < 0.001] but no significant effect for Group [*F*_(__1_,_55__)_ = 1.27, *p* = 0.265]. There was also no significant Session by Group interaction [*F*_(__1_,_55__)_ = 0.706, *p* = 0.404]. This means that both groups improved after the second session. *Post hoc* analyses revealed that both the control group (*p* = 0.002) as well as the experimental group (*p* = 0.002) improved from S1 to S2.

### Neuroimaging Results

**FIGURE 7:**
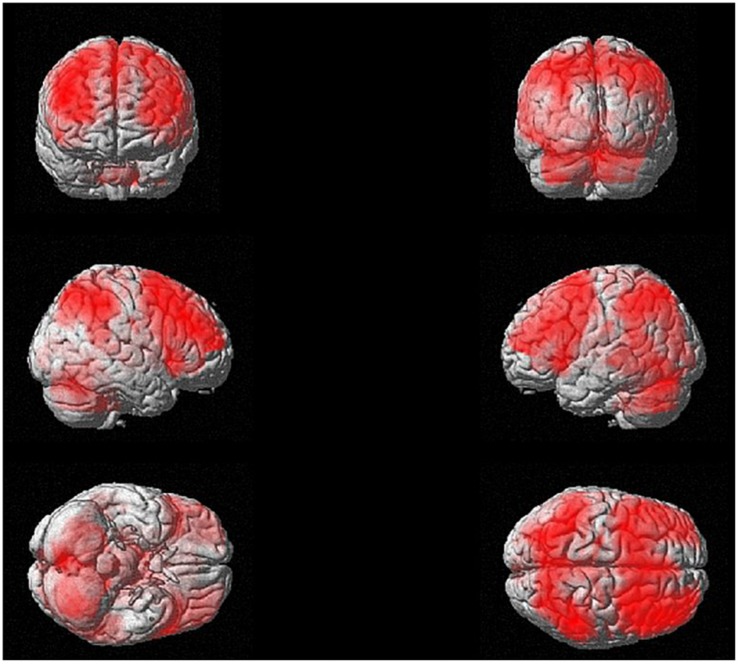
N-back activation at baseline (i.e., one-sample *t*-test for 3-back > X-back at *p* < 0.05 FDR corrected with a cluster extension of *k* = 53 voxels).

The whole-brain one-sample *t* test to investigate the brain regions activated in the n-back task (3-back > X-back) independent from training revealed wide-spread cortical as well as subcortical activity (Figure 7). We found activity mainly in bilateral precuneus, superior parietal lobule, inferior parietal lobule, superior frontal gyrus, sub-gyral frontal lobe, medial frontal gyrus, cingulate gyrus, and different parts of the cerebellum. There was also activity in the thalamus, specifically in the medial dorsal nucleus and in subcortical regions such as insula and caudate. These results were *p* < 0.05 FDR corrected with a cluster extension of *k* = 53 voxels.

We also performed a two-sample *t* test at S1 to investigate whether there were any baseline differences between the experimental and the active control group in the n-back task (3-back > X-back). The analysis yielded no significant differences. This means that we can interpret the differences between the groups at S2 as differences arising from the training. All results were *p* < 0.05 FDR corrected.

**FIGURE 8:**
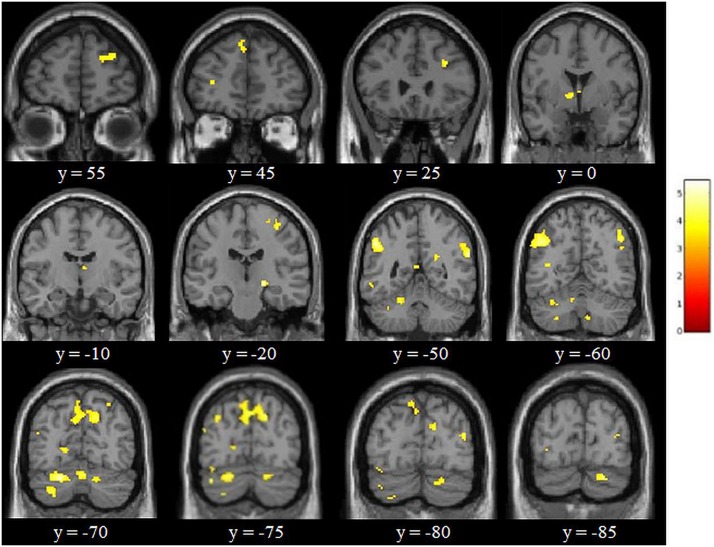
Results of the adaptive online n-back training [i.e., experimental group (S1 > S2) > control group (S1 > S2) for 3-back > X-back at *p* < 0.05 FDR corrected with a cluster extension of *k* = 6 voxels). Coordinates are in MNI space and the color bar expresses the *t*-score.

The factorial repeated-measures ANOVAs with Group (experimental group vs. control group) as between-subjects factor and Session (S1 vs. S2) as within-subject factor investigating the effects of the cognitive training in both groups for 3-back vs. X-back showed significant results for the interaction *Experimental Group (S1* > *S2)* > *Control Group (S1* > *S2)* in mainly superior frontal and parietal regions (see Table 2). The reverse contrast did not yield any significant results. In addition, the comparison *Experimental Group S1* > *Experimental Group S2* yielded significant activation in mainly cerebellum and parietal regions (supramarginal gyrus) (see Table 3 and Figure 8). The reverse contrast did not yield any significant results indicating that there was a reduction of activity in specific brain regions in the experimental group after the training. The *Control Group S1* > *Control Group S2* as well as the *Control Group S1* < *Control Group S2* contrast did not show any significant results. All results were *p* < 0.05 FDR corrected.

**TABLE 2:** List of higher brain activation in the experimental group at S1 compared to S2 [i.e., experimental group (S1) > experimental group (S2) at *p* < 0.05 FDR corrected with a cluster extension of *k* = 10 voxels].

			**MNI space**			
**Name**	**BA**	**Cluster extent**	***x***	***y***	***z***	***Z*-value**
L. Cerebellum (Tuber)	–	25	−42	−76	−30	5.11
R. Substantia nigra	–	48	20	−20	−6	4.99
R. Supramarginal gyrus	40	294	60	−48	26	4.9
L. Supramarginal gyrus	40	533	−50	−50	38	4.81
L. Cerebellum (Uvula)	–	240	−22	−72	−26	4.79
L. Middle temporal gyrus	20	66	−56	−36	−8	4.65
R. Cingulate gyrus	31	40	22	−52	24	4.3
R. Cuneus	7	441	16	−72	38	4.27
R. Posterior cingulate	23	22	4	−30	26	4.22
R. Middle occipital gyrus	19	24	42	−78	16	4.21
L. Lentiform nucleus	–	24	−12	4	−2	4.18
L. Cerebellum (Uvula)	–	116	22	−84	−26	4.13
L. Cerebellum (Tonsil)	–	105	−28	−58	−48	4.12
L. Lingual gyrus	19	50	−18	−66	6	4.12
R. Cerebellum (Declive of Vermis)	–	58	0	−70	−22	4.11
R. Middle frontal gyrus	9	35	32	26	30	4.09
L. Cerebellum (Culmen)	–	27	−22	−50	−24	4.09
R. Cerebellum (Tonsil)	–	33	8	−64	−42	4.01
R. Paracentral lobule	5	57	20	−30	54	3.97
L. Cerebellum (Declive)	–	26	−40	−78	−16	3.93
R. Middle frontal gyrus	6	12	36	20	40	3.80
L. Posterior cingulate	29	40	−2	−48	12	3.78
R. Precentral gyrus	4	29	34	−20	58	3.78
L. Middle frontal gyrus	10	12	−32	44	10	3.78
R. Superior frontal gyrus	9	28	28	56	24	3.75
R. Superior parietal lobe	7	31	26	−66	52	3.73
L. Superior occipital gyrus	19	20	−34	−74	36	3.72
L. Inferior occipital gyrus	18	11	−34	−88	−2	3.69
R. Cuneus	18	19	12	−80	26	3.65
R. Cerebelleum (Culmen)	–	13	6	−38	0	3.65
R. Inferior parietal lobule	40	23	52	−30	36	3.65
R. Thalamus	–	17	4	−2	6	3.62
R. Middle occipital gyrus	18	10	32	−86	12	3.6
L. Middle temporal gyrus	37	12	−38	−60	12	3.59
L. Middle temporal gyrus	39	10	−48	−72	22	3.59
R. Thalamus	–	14	6	−12	12	3.55
R. Cerebellum (Culmen)	–	18	36	−52	−28	3.52
L. Superior frontal gyrus	8	21	−4	46	42	3.52
L. Parahippocampal gyrus	30	12	−12	−42	4	3.51

*L, left; R, right; BA, brodmann area.*

**TABLE 3:** List of brain activations for the interaction [i.e., experimental group (S1 > S2) > control group (S1 > S2) at *p* < 0.05 FDR corrected with a cluster extension of *k* = 6 voxels].

			**MNI space**			
**Name**	**BA**	**Cluster extent**	***x***	***y***	***z***	***Z*-value**
R. Cerebellum posterior lobe (declive)	–	29	0	−68	−22	5.07
L. Cerebellum posterior lobe (crus I)	–	14	−42	−76	−30	4.79
R. Substantia nigra	–	28	18	−22	−6	4.66
L. Middle temporal gyrus	20	8	−58	−38	−8	4.65
R. Cerebellum posterior lobe (tonsil)	–	10	16	−66	−34	4.44
L. Cerebellum posterior lobe (inferior semi-lunar)	–	87	−22	−76	−36	4.43
R. Middle occipital gyrus	19	9	40	−80	18	4.33
R. Angular gyrus	39	16	46	−58	40	4.33
L. Middle temporal gyrus	39	25	−52	−70	26	4.23
R. Superior frontal gyrus	9	11	18	50	28	4.23
R. Middle frontal gyrus	9	11	32	28	30	4.13
R. Superior frontal gyrus	9	7	18	56	24	4.09
L. Supramarginal gyrus	40	19	−50	−50	38	4.08
L. Parahippocampal gyrus	30	7	−12	−42	6	4.03
R. Supramarginal gyrus	40	15	60	−46	24	4.02
L. Middle temporal gyrus	39	27	−48	−62	38	4.00
R. Posterior cingulate	29	7	4	−44	10	3.97
R. Anterior cingulate	32	7	6	46	4	3.93
L. Cuneus	7	6	−2	−72	40	3.91
R. Occipital lobe (cuneus)	18	7	12	−80	24	3.9

*L, left; R, right; BA, brodmann area.*

## Discussion

In the present study, we applied task-fMRI to investigate neural and behavioral effects of an 8-week adaptive online vWM training in middle-aged healthy subjects. We found no differences in brain activity during the n-back task between the experimental and active control group at baseline. Comparing both time points the results showed no activation differences in the control group, but a significantly decreased activation in vWM characteristic regions in the experimental group after the training. These activation decreases, most probably reflecting training-associated gains in cerebral efficiency, were accompanied by significant vWM performance improvements in the experimental group.

### Pre-training Activation

The general (i.e., training-independent) activation in a predominantly fronto-parieto-cerebellar network that we found by analyzing activation of the whole group at the first timepoint is largely in line with previous studies investigating vWM (Owen et al., 2005; Rottschy et al., 2012; Emch et al., 2019). However, one aspect which seems to distinguish the present results from previous findings especially in, on average, younger populations is the rather bilateral prefrontal activation in the present study (Cabeza, 2002; Cabeza et al., 2004). This weakly lateralized activity in predominantly frontal areas speaks in favor of the hemispheric asymmetry reduction in older adults (HAROLD) model (Cabeza, 2002) stating that lateralization/specialization in brain activity decreases with increasing age. There are different hypotheses regarding the underlying mechanism. One hypothesis assumes a compensatory mechanism underlying this activity expansion, whereas another assumption suggests a less specific recruitment of neural networks due to gradual changes that happen with age. Even though the present findings do not allow drawing any conclusions on the mechanism explaining this phenomenon, they nevertheless provide additional support in favor of this model.

### Training-Related Changes in Activation

Adaptive online vWM training resulted in reduced brain activity in several parietal areas, first and foremost in the left supramarginal gyrus (SMG), which has been found to be important for the phonological store component, although the exact neural basis of this WM component is still under debate (Buchsbaum and D’Esposito, 2008; Aboitiz et al., 2010). We also found reduced activation in the right homologous region. The right SMG has also been reported to be engaged during vWM in a study by Deschamps et al. (2014). When inhibiting activation of the SMG by applying TMS on both sides participants had a slower performance in the verbal 2-back task – an indicator for the involvement of the bilateral SMG in vWM. We also found decreased activation in a number of additional frontal, parietal and cerebellar regions, and thus in regions known to closely interplay in any kind of WM task (Owen et al., 2005; Rottschy et al., 2012; Emch et al., 2019). Surprisingly, there was also a decreased activation in the right substantia nigra, which supports the previously discussed hypothesis that this region is not only crucial for motor functions but also involved in learning and memory functions (Packard and Knowlton, 2002). Moreover, decreased activation in the experimental group after the training was detectable in the middle temporal gyrus. In a study with chronically intractable epilepsy patients this region has been found to represent stimuli held in WM (Kornblith et al., 2017). While up to the publication of this study the role of the middle temporal gyrus in WM processes was controversial, it is assumed to play a central role in the temporary maintenance of stimuli in WM. In addition, there was a reduced activity in the bilateral posterior cingulate gyrus, which is robustly activated during vWM tasks as demonstrated in our recently published meta-analysis (Emch et al., 2019), as well as in the bilateral cuneus, which has been reported to be activated with increasing memory load in vWM (Habeck et al., 2012).

These results are consistent with previous neuroimaging studies that show decreased activation in regions involved in WM processing following cognitive training (Schneiders et al., 2012; Schweizer et al., 2013; Miró-Padilla et al., 2018). Critically, none of these studies included an active control group. Hence, although the findings of these studies are relevant, it remains somewhat unknown whether the reported training effects were specific to WM or to the training itself, regardless of the type of training. Conversely, a study by Thompson et al. (2016) studied WM training effects with an active control group. Their experimental group performed WM training with a dual n-back task, the active control group performed a similarly intensive visuospatial training task demanding multiple objects tracking whereas the passive control group did not participate in any training but merely performed the same n-back task as the other groups before, and after the WM training time interval. They found that the experimental group compared to the active control group exhibited significantly reduced brain activity at 2-back and 3-back conditions in WM characteristic fronto-parietal networks. Vartanian et al. (2013) performed a study to investigate the effects of a verbal n-back training on a classical test of divergent thinking. Participants in the active control group completed a 4-choice RT task. The experimental group showed activity reductions in specific regions of the prefrontal cortex. Brehmer et al. (2011) examined the neural activity following 5 weeks of intensive WM training in healthy older adults. Similar to our design, in this study the experimental group received an adaptive training whereas the active control group did a fixed low-level practice. They did not find specific training-related changes in WM but the experimental group showed a larger decrease in cortical brain regions compared to the active control group in a high load WM task. As mentioned before, given methodological differences between studies, results on WM training effects are still heterogeneous with some studies also reporting training-associated increases in activation (Salmi et al., 2018). Nevertheless, our findings and the results of methodologically similar studies led us to conclude that the decreased activation in WM areas after training can be interpreted as an indicator of a training-associated increase in neural efficiency (i.e., less neural energy needs to be invested in order to attain the same or an even better performance level after training). In other terms, practice-related activation decreases are the result of a more efficient use of specific neuronal circuits (Poldrack, 2000; Kelly and Garavan, 2005). This assumption is supported by a couple of additional aspects. First, studies demonstrating a negative association between WM activation and performance -i.e., with better performing subjects showing less activation in WM-characteristic networks (Bokde et al., 2010; Zilles et al., 2016)- reinforce this hypothesis. Second, the above mentioned HAROLD model is based on this assumption. According to this model younger people, usually characterized by higher cognitive capacities, tend to demonstrate less (i.e., more restricted, more lateralized) activation in relevant networks compared to elderly people. Third, findings showing a linear relationship between vWM demands and activation in WM-relevant regions clearly illustrate an association between the level of cognitive demand and the strength and extent of neural activation (Champod and Petrides, 2010). Also, our results are somewhat consistent with the CRUNCH theory, which stands for the “compensation-related utilization of neural circuit’s hypothesis” (Reuter-Lorenz and Cappell, 2008). It suggests that older adults engage more neural activity than younger adults to meet task demands. The brain activity reduction after training in the experimental group may be explained by this theory, since after the training this group activated less brain regions in order to perform the vWM task successfully. We could hypothesize that after training the brain activity of older adults during the task is more similar to a “younger brain,” potentially as a result of neural plasticity. Thus, we assume that the decreased activation after training in association with decreasing WM demands (i.e., in our study as a result of intensive WM training) reflects a higher neuro-cognitive efficiency brought about by the vWM training.

### Behavioral Changes and Direct Effects

As expected, the training-associated changes in neural activation were accompanied by a significant enhancement in vWM performance in the fMRI task. Thus, we observed a significant improvement in the experimental group in terms of *d’* values for the vWM condition (i.e., 3-back condition) whereas there was no such improvement in the low-level X-back condition demanding merely attentional processes. Considering that the training was an adaptive WM training this result is according to expectation. Interestingly, mean reaction times in the 3-back condition decreased in both groups, with the experimental group, however, improving to a considerably larger extent. Taking into account that motor response was practiced in both trainings, this result is likewise in line with our expectations. The performance improvement in the vWM condition from the fMRI task (i.e., 3-back level) in the experimental group was backed up by a significant training performance improvement of this group. This means that the improvement manifested itself both in the n-back task performed on the home-computer as well as in a different environment (i.e., in the fMRI scanner) with a stable n-back level - a clear indication of practice effects. Moreover, the experimental group improved their HAWIE-R digit span forward (i.e., vWM) performance compared to the control group thus demonstrating direct effects on a similar vWM task. Hence, the training had the expected effects on vWM performance. These results imply that the training was an effective and adequate method to improve WM-relevant processes (i.e., the encoding, maintenance, and retrieval of verbal stimulus material). The finding that there were no significant improvements in the digit span backward test could be due to the fact that this subtest is significantly more complex than the forward version. Considering that the vWM training did not possess this level of complexity the lacking significance in the backward version is in line with recent results suggesting that the effects of WM training tend to be restricted to the cognitive demands provided by the training (Holmes et al., 2019; Linares et al., 2019).

Findings from previous studies seem to largely corroborate the effectiveness of WM trainings. Thus, Dahlin et al. (2008) examined the effects of a 5-week computer-based training demanding information updating in WM in a group of young and older adults. They observed significant training gains in both groups with the younger adults, however, recalling more four-letter sequences compared to the older trainees. Another study by Li et al. (2008) examined the effects of a 45-day non-adaptive spatial n-back training both in younger and older adults. Both groups improved in a spatial and a numerical 3-back task as well as in additional WM tasks. Similar results were reported by Buschkuehl et al. (2008). In a senior cohort they investigated the effects of a WM training which consisted of three tasks: one simple and two complex WM span tasks. As opposed to Dahlin et al. (2008) and Li et al. (2008), they investigated an active control group participating in light physical training. They also reported significant improvements on the training tasks in the experimental group compared to the active control group. In a study by Brehmer et al. (2012) two groups of participants (a younger and an older cohort) were investigated. Half of them performed an adaptive training, the other half performed a low-level task difficulty training (i.e., active condition). Their results indicated that the adaptive training led to larger training gains compared to the low-level practice, even in the older cohort. The results by Brehmer et al. (2012) are moreover in line with another recent study demonstrating an increase in WM performance in older individuals as a consequence of an adaptive computerized WM training (Tusch et al., 2016). Taken together, these findings and the results from our study suggest that there is room for cognitive improvement also at advanced age.

### Limitations

This study has some limitations. First, the control group performed a fixed n-back level during the 32 sessions not allowing them to improve. The training was too easy for them and we see a ceiling effect because most active control participants achieved the highest possible scores in a short period of time. This means that there is little or no variance between the participants – a fact which complicated result interpretation. Second, we did not control for lure items in the adaptive online n-back training. Lure items in the n-back task are non-target items that match an item earlier in the sequence but not at the current critical target position (Oberauer, 2005). Participants could potentially have responded to the item not because of the specific location but because of familiarity, leading to this interference. This problem is particularly pronounced among older adults suggesting that the contribution of familiarity items to WM performance increases with age (Schmiedek et al., 2009). Future studies should take these limitations into account. Nevertheless, we think that this paper helps us to understand how WM training can lead to an improved neural efficiency in middle-aged adults.

## Conclusion

The present vWM training study which was carefully designed by taking into account methodologically relevant influencing factors (i.e., active control group, performance adapted training design, feedback during the training to motivate the participants, and advanced-age participants with a limited age range) led to significant activation decreases in WM-relevant regions and considerable improvements in vWM performance. In correspondence with the concept of “lifelong learning” present results clearly indicate that neural plasticity and behavioral improvement following vWM training is possible not only at younger age, but also in middle-aged adults.

## Data Availability Statement

The raw data supporting the conclusions of this manuscript will be made available by the authors, without undue reservation, to any qualified researcher.

## Ethics Statement

The studies involving human participants were reviewed and approved by the Ethical Committee of the Klinikum Rechts der Isar. The patients/participants provided their written informed consent to participate in this study.

## Author Contributions

ME, IY, and KK contributed to the conception and design of the study. ME designed the stimuli and online training, programed the tasks, analyzed the neuroimaging data and training data, and wrote the first draft of the manuscript. ME and IR recruited, scanned, and tested the participants. IR analyzed part of the task-fMRI behavioral data and revisited all different versions of the manuscript. QW analyzed the cognitive tests. KK wrote sections of the manuscript. All authors contributed to the manuscript revision and approved the submitted version.

## Conflict of Interest

The authors declare that the research was conducted in the absence of any commercial or financial relationships that could be construed as a potential conflict of interest.

## Footnotes

1 https://jasp-stats.org/

2 www.neurobs.com

3 http://www.fil.ion.ucl.ac.uk/spm

## References

[B1] Aboitiz F. Aboitiz S. García R. R. (2010). The phonological loop: a key innovation in human evolution. *Curr. Anthropol.* 51 S55–S65. 10.1086/650525

[B2] Ashburner J. (2007). A fast diffeomorphic image registration algorithm. *Neuroimage* 38 95–113. 10.1016/j.neuroimage.2007.07.007 17761438

[B3] Baddeley A. (2010). Working memory. *Curr. Biol.* 20 136–140. 10.1016/j.cub.2009.12.014 20178752

[B4] Berit A. Ove D. (1998). The clock-drawing test. *Age Aging* 27 399–403. 10.1093/ageing/afs149 23144287

[B5] Bokde A. Karmann M. Born C. Teipel S. Omerovic M. Ewers M. (2010). Altered brain activation during a verbal working memory task in subjects with amnestic mild cognitive impairment. *J. Alzheimers Dis.* 21 103–118. 10.3233/JAD-2010-091054 20413893

[B6] Borella E. Carretti B. Riboldi F. De Beni R. (2010). Working memory training in older adults: evidence of transfer and maintenance effects. *Psychol. Aging* 25 767–778. 10.1037/a0020683 20973604

[B7] Brehmer Y. Rieckmann A. Bellander M. Westerberg H. Fischer H. Bäckman L. (2011). Neural correlates of training-related working-memory gains in old age. *Neuroimage* 58 1110–1120. 10.1016/j.neuroimage.2011.06.079 21757013

[B8] Brehmer Y. Westerberg H. Bäckman L. (2012). Working-memory training in younger and older adults: training gains, transfer, and maintenance. *Front. Hum. Neurosci.* 6:63. 10.3389/fnhum.2012.00063 22470330 PMC3313479

[B9] Buchsbaum B. R. D’Esposito M. (2008). The search for the phonological store: from loop to convolution. *J. Cogn. Neurosci.* 20 762–778. 10.1162/jocn.2008.20501 18201133

[B10] Buschkuehl M. Jaeggi S. M. Hutchison S. Perrig-Chiello P. Däpp C. Müller M. (2008). Impact of working memory training on memory performance in old-old adults. *Psychol. Aging* 23 743–753. 10.1037/a0014342 19140646

[B11] Cabeza R. (2002). Prefrontal and medial temporal lobe contributions to relational memory in young and older adults. *Psychol. Aging* 17 85–100. 10.1037//0882-7974.17.1.85 11931290

[B12] Cabeza R. Daselaar S. M. Dolcos F. Prince S. E. Budde M. Nyberg L. (2004). Task-independent and task-specific age effects on brain activity during working memory, visual attention and episodic retrieval. *Cereb. Cortex* 14 364–375. 10.1093/cercor/bhg133 15028641

[B13] Carretti B. Borella E. Zavagnin M. De Beni R. (2011). Impact of metacognition and motivation on the efficacy of strategic memory training in older adults: analysis of specific, transfer and maintenance effects. *Arch. Gerontol. Geriatr.* 52 e192–e197. 10.1016/j.archger.2010.11.004 21126778

[B14] Champod A. S. Petrides M. (2010). Dissociation within the frontoparietal network in verbal working memory: a parametric functional magnetic resonance imaging study. *J. Neurosci.* 30 3849–3856. 10.1523/JNEUROSCI.0097-10.2010 20220020 PMC6632229

[B15] Chooi W. (2012). Working memory and intelligence: a brief review. *J. Educ. Develop. Psychol.* 2 42–50. 10.5539/jedp.v2n2p42

[B16] Dahlin E. Nyberg L. Bäckman L. Neely A. S. (2008). Plasticity of executive functioning in young and older adults: immediate training gains, transfer, and long-term maintenance. *Psychol. Aging* 23 720–730. 10.1037/a0014296 19140643

[B17] Daneman A. Carpenter P. A. (1980). Individual differences in working memory and reading. *J. Verbal Learn. Verbal Behav.* 19 450–466. 10.1016/S0022-5371(80)90312-6

[B18] Deschamps I. Baum S. R. Gracco V. L. (2014). Neuropsychologia on the role of the supramarginal gyrus in phonological processing and verbal working memory: evidence from rTMS studies. *Neuropsychologia* 53 39–46. 10.1016/j.neuropsychologia.2013.10.015 24184438

[B19] Emch M. von Bastian C. C. Koch K. (2019). Neural correlates of verbal working memory: an fMRI meta-analysis. *Front. Hum. Neurosci.* 13:180. 10.3389/fnhum.2019.00180 31244625 PMC6581736

[B20] Folstein M. F. Folstein S. E. McHugh P. R. (1975). Mini-mental state”. a practical method for grading the cognitive state of patients for the clinician. *J. Psychiatr. Res.* 12 189–198. 1202204 10.1016/0022-3956(75)90026-6

[B21] Haatveit B. C. Sundet K. Hugdahl K. Ueland T. Melle I. Andreassen O. A. (2010). The validity of d prime as a working memory index: results from the bergen n-back task. *J. Clin. Exp. Neuropsychol.* 32 871–880. 10.1080/13803391003596421 20383801

[B22] Habeck C. Rakitin B. Stefener J. Stern Y. (2012). Contrasting visual working memory for verbal and non-verbal material with multivariate analysis of fMRI. *Brain Res.* 1467 27–41. 10.1016/j.brainres.2012.05.045.Contrasting 22652306 PMC3398171

[B23] Holmes J. Woolgar F. Hampshire A. Gathercole S. E. Holmes J. (2019). Are working memory training effects paradigm-specific. *Front. Psychol.* 10:1103. 10.3389/fpsyg.2019.01103 31178781 PMC6542987

[B24] Jaeggi S. M. Buschkuehl M. Jonides J. Perrig W. J. (2008). Improving fluid intelligence with training on working memory. *Proc. Natl. Acad. Sci. U.S.A.* 105 6829–6833. 10.3758/s13423-014-0699-x 18443283 PMC2383929

[B25] Jaeggi S. M. Studer-Luethi B. Buschkuehl M. Su Y. F. Jonides J. Perrig W. J. (2010). The relationship between n-back performance and matrix reasoning - implications for training and transfer. *Intelligence* 38 625–635. 10.1016/j.intell.2010.09.001

[B26] Jansma J. M. Ramsey N. F. Slagter H. A. Kahn R. S. (2001). Functional anatomical correlates of controlled and automatic processing. *J. Cogn. Neurosci.* 13 730–743. 10.1162/08989290152541403 11564318

[B27] Kelly A. M. C. Garavan H. (2005). Human functional neuroimaging of brain changes associated with practice. *Cereb. Cortex* 15 1089–1102. 10.1093/cercor/bhi005 15616134

[B28] Klingberg T. Forssberg H. Westerberg H. (2002). Training of working memory in children with ADHD. *J. Clin. Exp. Neuropsychol.* 24 781–791. 10.1076/jcen.24.6.781.8395 12424652

[B29] Knopman D. S. (2012). Subjective cognitive impairment. *Neurology* 79 1308–1309. 10.1212/WNL.0b013e31826c1bd1 22914836

[B30] Kornblith S. Quiroga R. Q. Koch C. Fried I. Mormann F. Kornblith S. (2017). Persistent single-neuron activity during working memory in the human medial temporal lobe. *Curr. Biol.* 27 1026–1032. 10.1016/j.cub.2017.02.013 28318972 PMC5510887

[B31] Kulikowski K. Potasz-Kulikowska K. (2016). Can we measure working memory via the Internet? the reliability and factorial validity of an online n-back task. *Pol. Psychol. Bull.* 47 51–61. 10.1515/ppb-2016-0006

[B32] Landsberger H. A. (1958). Hawthorne revisited. *Soc. Forces* 37:119.

[B33] Li S. C. Schmiedek F. Huxhold O. Röcke C. Smith J. Lindenberger U. (2008). Working memory plasticity in old age: practice gain, transfer, and maintenance. *Psychol. Aging* 23 731–742. 10.1037/a0014343 19140644

[B34] Linares R. Borella E. Lechuga M. T. Carretti B. Pelegrina S. (2019). Nearest transfer effects of working memory training: a comparison of two programs focused on working memory updating. *PLoS One* 14:e0211321. 10.1371/journal.pone.0211321 30759135 PMC6373913

[B35] Meule A. (2017). Reporting and interpreting working memory performance in n-back tasks. *Front. Psychol.* 8:352. 10.1002/hup.1248 28326058 PMC5339218

[B36] Miró-Padilla A. Bueichekú E. Ventura-campos N. (2018). Long-term brain effects of N-back training: an fMRI study. *Brain Imaging Behav.* 13 1115–1127. 10.1007/s11682-018-9925-x 30006860

[B37] Molz G. Schulze R. Schroeders U. Wilhelm O. (2010). Wechsler intelligenztest für erwachsene WIE. deutschsprachige bearbeitung und adaptation des WAlS-lI! von david wechsler. *Psychol. Rundsch.* 61 229–230./a000042

[B38] Oberauer K. (2005). Binding and inhibition in working memory: individual and age differences in short-term recognition. *J. Exp. Psychol.* 134 368–387. 10.1037/0096-3445.134.3.368 16131269

[B39] Owen A. M. McMillan K. M. Laird A. R. Bullmore E. (2005). N-back working memory paradigm: a meta-analysis of normative functional neuroimaging studies. *Hum. Brain Mapp.* 25 46–59. 10.1002/hbm.20131 15846822 PMC6871745

[B40] Packard M. G. Knowlton B. J. (2002). Learning and memory functions of the basal ganglia. *Annu. Rev. Neurosci.* 25 563–593. 10.1146/annurev.neuro.25.112701.142937 12052921

[B41] Park D. C. Reuter-Lorenz P. (2009). The adaptive brain: aging and neurocognitive scaffolding. *Annu. Neurosci.* 60 173–196. 10.1146/annurev.psych.59.103006.093656 PMC3359129 19035823

[B42] Pliatsikas C. Verissimo J. Babcock L. Pullman M. Y. Glei D. A. Weinstein M. (2018). Working memory in older adults declines with age, but is modulated by sex and education. *Q. J. Exp. Psychol.* 72 1308–1327. 10.1177/1747021818791994 30012055

[B43] Poldrack R. A. (2000). Imaging brain plasticity: conceptual and methodological issues — a theoretical review. *Neuroimage* 12 1–13. 10.1006/nimg.2000.0596 10875897

[B44] Power J. D. Barnes K. A. Snyder A. Z. Schlaggar B. L. Petersen S. E. (2012). Spurious but systematic correlations in functional connectivity MRI networks arise from subject motion. *Neuroimage* 59 2142–2154. 10.1016/j.neuroimage.2011.10.018 22019881 PMC3254728

[B45] Power J. D. Mitra A. Laumann T. O. Synder A. Z. Schlaggar B. L. Petersen S. E. (2014). Methods to detect, characterize, and remove motion artifact in resting state fMRI. *Neuroimage* 84 1–45. 10.1016/j.neuroimage.2013.08.048.Methods 23994314 PMC3849338

[B46] Reuter-Lorenz P. A. Cappell K. A. (2008). Neurocognitive aging and the compensation hypothesis. *Curr. Direct. Psychol. Sci.* 17 177–182. 10.1111/j.1467-8721.2008.00570.x

[B47] Rottschy C. Langner R. Dogan I. Reetz K. Laird A. R. Schulz J. B. (2012). Modelling neural correlates of working memory: a coordinate-based meta-analysis. *Neuroimage* 60 830–846. 10.1016/j.neuroimage.2011.11.050 22178808 PMC3288533

[B48] Salmi J. Nyberg L. Laine M. (2018). Working memory training mostly engages general-purpose large-scale networks for learning. *Neurosci. Biobehav. Rev.* 93 108–122. 10.1016/j.neubiorev.2018.03.019 29574197

[B49] Schmiedek F. Hildebrandt A. Lövdén M. Wilhelm O. Lindenberger U. (2009). Complex span versus updating tasks of working memory: the gap is not that deep. *J. Exp. Psychol.* 35 1089–1096. 10.1037/a0015730 19586272

[B50] Schneiders J. A. Opitz B. Tang H. Deng Y. Xie C. Li H. (2012). The impact of auditory working memory training on the fronto-parietal working memory network. *Front. Hum. Neurosci.* 6:173. 10.3389/fnhum.2012.00173 22701418 PMC3373207

[B51] Schweizer S. Grahn J. Hampshire A. Mobbs D. Dalgleish T. (2013). Training the emotional brain: improving affective control through emotional working memory training. *J. Neurosci.* 33 5301–5311. 10.1523/JNEUROSCI.2593-12.2013 23516294 PMC6704999

[B52] Sheehan D. V. Lecrubier Y. Sheehan K. H. Amorim P. Janavs J. Weiller E. (1998). The mini-international neuropsychiatric interview (M.I.N.I.): the development and validation of a structured diagnostic psychiatric interview for DSM-IV and ICD-10. *J. Clin. Psychiatry* 59 22–57. 9881538

[B53] Siegel J. S. Power J. D. Dubis J. W. Vogel A. C. Church J. A. Schlaggar B. L. (2014). Statistical improvements in functional magnetic resonance imaging analyses produced by censoring high-motion data points. *Hum. Brain Mapp.* 35 1981–1996. 10.1002/hbm.22307 23861343 PMC3895106

[B54] Thompson T. W. Waskom M. L. Gabrieli J. D. E. (2016). Intensive working memory training produces functional changes in large-scale fronto-parietal networks. *J. Cogn. Neurosci.* 28 575–588. 10.1162/jocn 26741799 PMC5724764

[B55] Tusch E. S. Alperin B. R. Ryan E. Holcomb P. J. Mohammed A. H. Daffner K. R. (2016). Changes in neural activity underlying working memory after computerized cognitive training in older adults. *Front. Aging Neurosci.* 8:255. 10.3389/fnagi.2016.00255 27877122 PMC5099139

[B56] Vartanian O. Jobidon M. Bouak F. Nakashima A. Smith I. Lam Q. (2013). Working memory training is associated with lower prefrontal cortex activation in a divergent thinking task. *Neuroscience* 236 186–194. 10.1016/j.neuroscience.2012.12.060 23357116

[B57] von Bastian C. C. Oberauer K. (2014). Effects and mechanisms of working memory training: a review. *Psychol. Res.* 78 803–820. 10.1007/s00426-013-0524-6 24213250

[B58] Wiley J. Jarosz A. F. (2012). Working memory capacity, attentional focus, and problem solving. *Curr. Dir. Psychol. Sci.* 21 258–262. 10.1177/0963721412447622 19123118

[B59] Yesavage J. A. Brink T. L. Rose T. L. Lum O. Huang V. Adey M. (1983). Development and validation of a geriatric depression screening scale: a preliminary report. *J. Psychiatric Res.* 17 37–49. 10.1016/0022-3956(82)90033-4 7183759

[B60] Zilles D. Lewandowski M. Vieker H. Henseler I. Diekhof E. Melcher T. (2016). Gender differences in verbal and visuospatial working memory performance and networks. *Neuropsychobiology* 73 52–63. 10.1159/000443174 26859775

